# Smartphone Applications with Sensors Used in a Tertiary Hospital—Current Status and Future Challenges

**DOI:** 10.3390/s150509854

**Published:** 2015-04-27

**Authors:** Yu Rang Park, Yura Lee, Guna Lee, Jae Ho Lee, Soo-Yong Shin

**Affiliations:** 1 Clinical Research Center, Asan Medical Center, Seoul 138-736, Korea; E-Mail: yurang.park@amc.seoul.kr; 2 Department of Biomedical Informatics, Asan Medical Center, Seoul 138-736, Korea; E-Mails: haepary@naver.com (Y.L.); rufiji@gmail.com (J.H.L.); 3 Division of Nursing Science, College of Health Science, Ewha Womans University, Seoul 120-750, Korea; E-Mail: finside00@gmail.com; 4 Ubiquitous Health Center, Asan Medical Center, Seoul 138-736, Korea; 5 Department of Emergency Medicine, Asan Medical Center, University of Ulsan College of Medicine, Seoul 138-736, Korea; 6 Division of General Internal Medicine and Primary Care, Brigham and Women’s Hospital, Boston, MA 02467, USA

**Keywords:** healthcare app, data entry, mobile health, smartphone sensors

## Abstract

Smartphones have been widely used recently to monitor heart rate and activity, since they have the necessary processing power, non-invasive and cost-effective sensors, and wireless communication capabilities. Consequently, healthcare applications (apps) using smartphone-based sensors have been highlighted for non-invasive physiological monitoring. In addition, several healthcare apps have received FDA clearance. However, in spite of their potential, healthcare apps with smartphone-based sensors are mostly used outside of hospitals and have not been widely adopted for patient care in hospitals until recently. In this paper, we describe the experience of using smartphone apps with sensors in a large medical center in Korea. Among >20 apps developed in our medical center, four were extensively analyzed (“My Cancer Diary”, “Point-of-Care HIV Check”, “Blood Culture” and “mAMIS”), since they use smartphone-based sensors such as the camera and barcode reader to enter data into the electronic health record system. By analyzing the usage patterns of these apps for data entry with sensors, the current limitations of smartphone-based sensors in a clinical setting, hurdles against adoption in the medical center, benefits of smartphone-based sensors and potential future research directions could be evaluated.

## 1. Introduction

The number of global smartphone subscribers is expected to reach 3.5 billion by 2019 [1]. Due to their popularity, processing power, non-invasive and cost-effective sensors, and wireless communication capabilities, smartphones have received great attention in healthcare settings [2,3,4,5]. A smartphone was used by 74% of physicians for professional purposes in 2013 [4]. As a consequence, the mobile health (m-health) market has grown rapidly and will reach $49 billion by 2020 [6]. Patients and consumers also expect that m-health will improve the quality and convenience of healthcare by reducing costs [7]. Hence, many healthcare applications (apps) have now been developed and commercialized (*i.e.*, ECG monitoring and atrial fibrillation detection [8], eye disease detection [9]). Several apps have also received FDA clearance [10]. Additionally, in the United States, the Medical Electronic Data Technology Enhancement for Consumers’ Health (MEDTECH) Act was recently proposed to exempt low-risk medical software and mobile apps from FDA regulation [11].

Among the enormous number of healthcare apps available for smartphones, some apps use diverse sensors for data entry. The sensors for smartphones can be classified into two categories including smartphone-based sensors and add-on sensors. Smartphone-based sensors include the cameras, accelerometers, gyroscopes, magnetometers, proximity sensors, light sensors, barometers, thermometers, air humidity sensors, and pedometers which are integrated into most smartphones [12]. Some recent smartphones have additional biometric sensors including heart rate monitors and fingerprint sensors [12]. Add-on sensors use external devices such as smartphone cases [8]. Most apps with sensors have been highlighted for non-invasive physiological monitoring outside of hospitals. In spite of their promise, healthcare apps with sensors have not been widely adopted for patient care within hospitals until recently [3,13]. Most apps are designed for reference or education purposes only [13].

In our current study, we report our experiences of using smartphone apps with smartphone-based sensors in our hospital, the Asan Medical Center (AMC). AMC is the largest medical center in Korea with approximately 2700 inpatient beds and 10,000 outpatient visits per day. Since its establishment, the hospital information system has been actively used to improve quality of care and to make the clinical workflow more efficient [14]. In the early 2000s, the AMC began using m-health services such as a mobile electronic medical record (EMR) system to improve the accessibility, mobility, and efficiency of patient care. The AMC also established a ubiquitous health center in 2009 to promote its m-health service in the hospital [15]. Due to the very large number of patients and the active use of the m-health service, our cases may be helpful to other hospitals trying to adopt or expand m-health.

## 2. Materials and Methods

### 2.1. Selection of AMC Healthcare Apps

Among more than 20 healthcare apps which have been developed at the AMC [15], four apps—“My Cancer Diary”, “Point-of-Care HIV Check”, “Blood Culture” and “mAMIS (mobile Asan Medical Information System)”—were chosen due to their use of smartphone-based sensors for data entry. The remaining apps do not have the functionality to enter the data from a sensor, but only to provide the necessary information to users. Table 1 summarizes the characteristics of these four apps. “My Cancer Diary” was developed for patient use and other apps were designed for use by healthcare providers.

**Table 1:** Characteristics of the chosen apps.

	My Cancer Diary	Point-of-Care HIV Check	Blood Culture	mAMIS
Description	Personal health management application for cancer patients	Point-of-Care HIV check	Point-of-Care blood culture sampling application	Mobile electronic medical record system
Target users	Patients	Nurses	Physicians	Physicians and nurses
Period of usage	Since October 2012	Since September 2013	Since May 2012	Since September 2014 *
Type of sensor	Camera (barcode or QR code recognition)	Camera (barcode recognition and image acquisition)	Camera (barcode recognition)	Touch ID (Fingerprint recognition)
Purpose of sensor	Log-in	Patient identification, result image acquisition	Patient and blood sample identification	Log-in
Supported OS	Android, iOS	Android	Android, iOS	iOS (8.0 and up)

* After supporting Touch ID for iPhone 6 and later.

#### 2.1.1. My Cancer Diary

**Figure 1:**
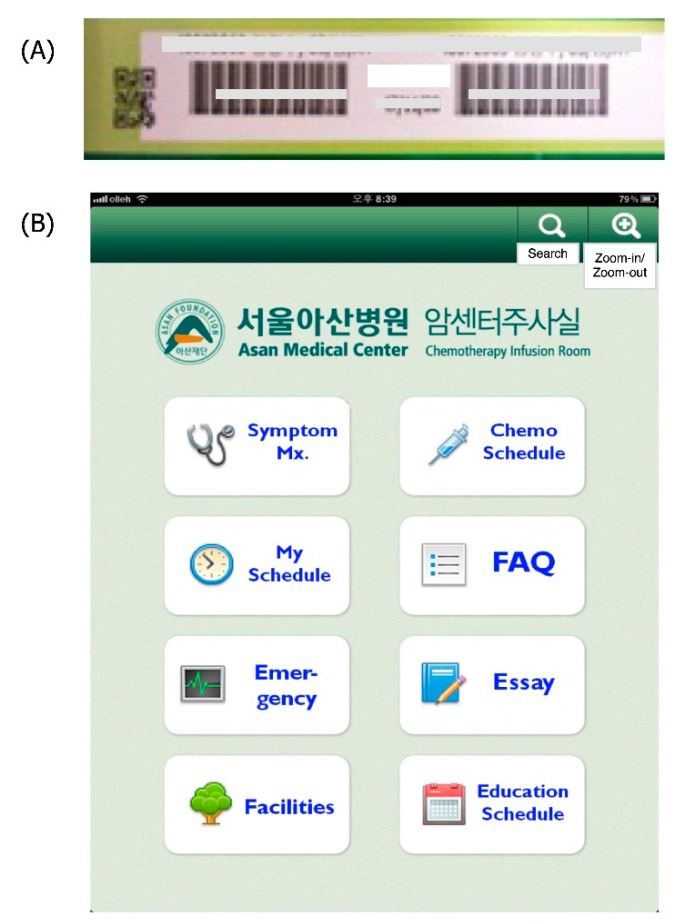
My Cancer Diary application (**A**) Patient barcode sample (**B**) Screenshot of main menu.

The “My Cancer Diary” app is a tool for cancer patient self-management. Usually, patient self-management tools provide information or functions such as general information about the disease, patient assistance, and healthcare professional assistance tools to aid in patient self-management of their chronic diseases or symptoms [16]. Following these trends, “My Cancer Diary” provides three types of information: (1) general information about cancer (anticancer drugs, serious symptoms, frequently asked questions about cancers, and patients’ essays on how they overcame their cancers); (2) a patient assistance tool (symptom management and my anticancer diary); and (3) healthcare professional assistance (cancer education). As mentioned in Table 1, “My Cancer Diary” used the camera to recognize the patient’s barcode or QR code for app login. Figure 1 shows the barcode login image (Figure 1A) and main menu of the app (Figure 1B).

#### 2.1.2. Point-of-Care HIV Check

Point-of-care diagnosis apps are essential to improve patient and care provider safety. A point-of-care diagnosis app in the AMC checks HIV infection of patients in the emergency room (ER) to improve care provider safety. In the ER, physicians and nurses contact many patients without previous laboratory studies to evaluate for possible infectious diseases, and 7% of occupationally acquired HIV infections occur in the ER [17,18]. For example, approximately 100,000 sharps injuries are reported from NHS hospitals in the United Kingdom. In the United States, approximately 400,000 sharps-related injuries among health care personnel are reported annually, and that number is considered an underestimate. Therefore, a fast and easily accessible report of the result of an HIV screening test is required to provide extra care to patients highly likely to be HIV-positive [19].

**Figure 2:**
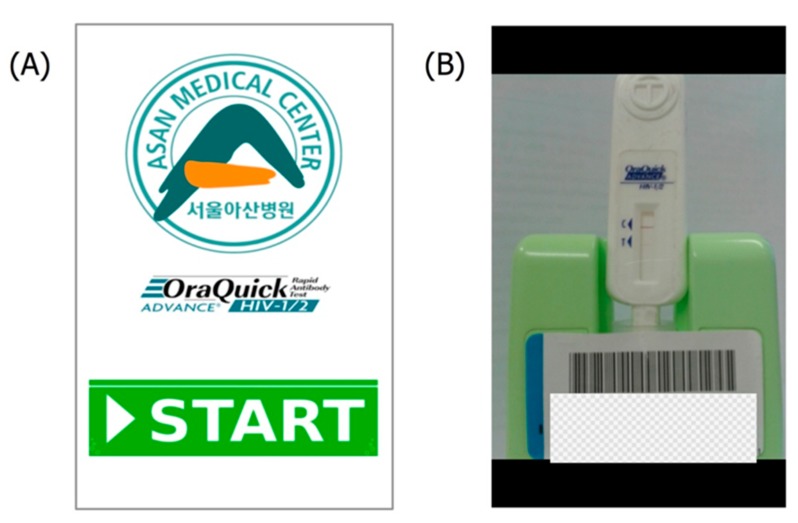
“Point-of-care HIV Check” application. (**A**) Screenshot of main menu; (**B**) Screenshot of barcode reading phase.

The developed “Point-of-Care HIV Check” app provides a local, rapid, and often lower-cost alternative to sending samples to a hospital laboratory for analysis [20]. The app identifies a patient’s ID using a barcode and takes a photo of the test results using the smartphone camera. Figure 2A shows the main menu of the app, and Figure 2B shows the barcode reading phase.

#### 2.1.3. Blood Culture

**Figure 3:**
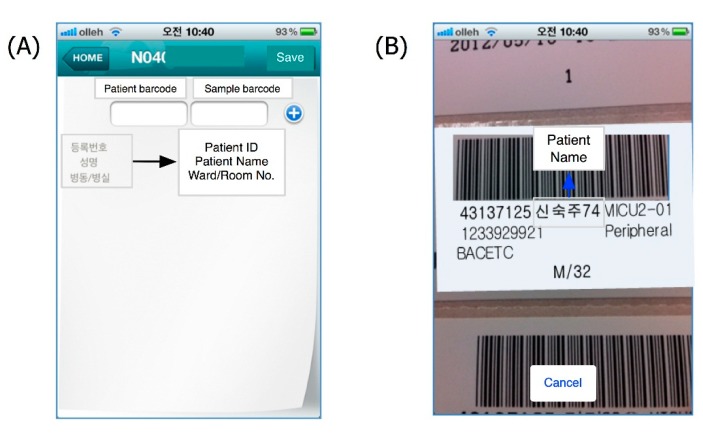

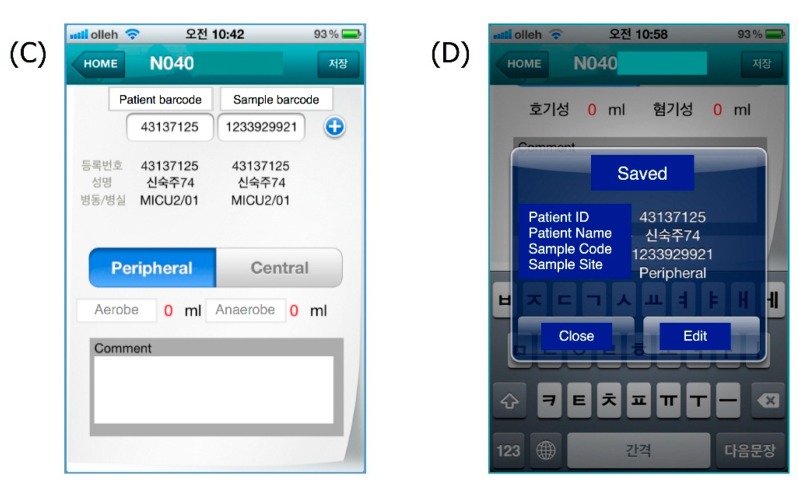
“Blood Culture” application. (**A**) Barcode reading menu; (**B**) Barcode reading phase; (**C**) Reading results and entering additional data such as the sample site and volume; (**D**) Saving data. In this figure, pseudonymized ID and patient name were used.

When physicians sample a patient’s blood for blood culture examination, they should sample the blood two or more times with a time gap between measurements [21]. The “Blood Culture” app was implemented to guide and alarm at the proper method and time to sample blood for culture. This app identifies the patient’s barcode using the smartphone camera and creates a timestamp for the blood culture [22]. Users must login to this app using a hospital staff ID and scan the patient and sample barcodes at each step (Figure 3A,B).

Afterwards, the sample site and volume can be entered manually (Figure 3C). These data are then transmitted to the legacy laboratory information system simultaneously (Figure 3D).

#### 2.1.4. mAMIS

**Figure 4:**
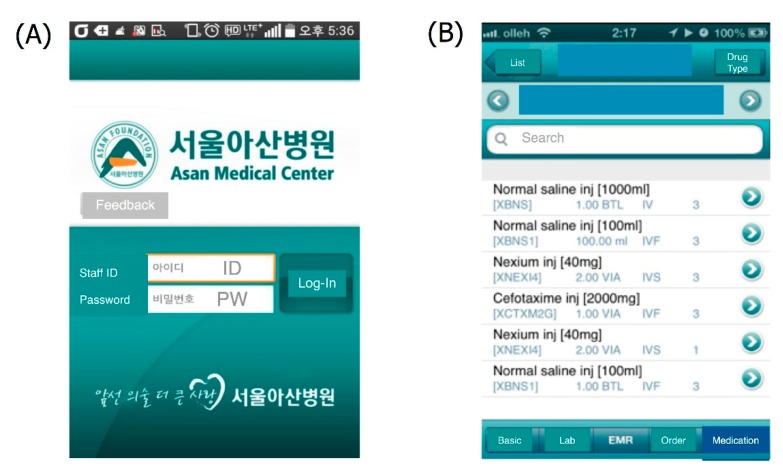

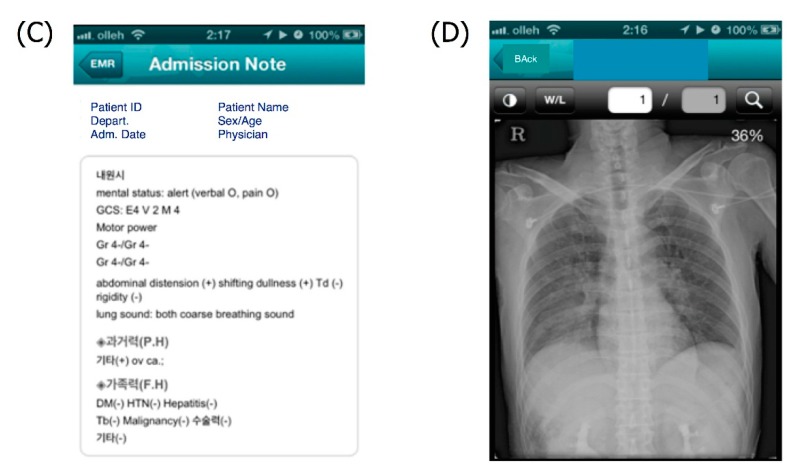
mAMIS application. (**A**) mAMIS login menu; (**B**) Medication information for the chosen patient; (**C**) Admission note; (**D**) PACS image.

mAMIS is a mobile EMR app which provides all medical records including medications, laboratory results, and images of radiologic studies completed since 2004 (Figure 4).

The app has been widely used since 2010, and a sensor for fingerprint recognition was added in September 2014. After introduction of Touch ID into Apple iPhone 6, the AMC developed the login capability of the mAMIS iOS version due to the large number of requests received.

### 2.2. Collection and Analysis of Usage Data

To check the current status of mobile sensor usage of healthcare apps in the AMC, the log data of each app were collected. Since all of the chosen apps were developed in-house, the necessary log data was easily extracted from the mobile application server or hospital information system server. The log data from the apps’ launch until November 2014 were collected for “My Cancer Diary”, “Point-of-Care HIV Check” and “Blood Culture” apps. The log data of “My Cancer Diary” was collected for 26 months, “Point-of-Care HIV Check” for 14 months, and “Blood Culture” for 29 months. The log data for “mAMIS” was collected from the app upgrade to support Touch ID (September 2014) until March 2015. Since the other apps have more than one year log data, we expanded the log data collection period for mAMIS (seven months). To represent the trend of mobile sensor usage of the four apps, we analyzed the log data using linear regressions and added the results as trend lines in the figures.

### 2.3. System Architecture of AMC Mobile Applications

**Figure 5:**
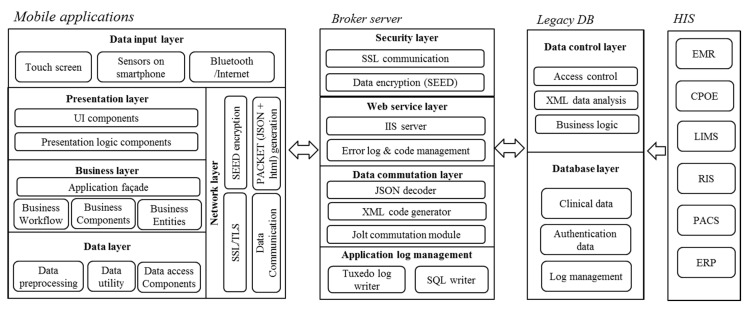
Technical architecture of mobile sensor applications. JSON: JavaScript Object Notation, SSL: Secure Socket Layer, TLS: Transport Layer Security, IIS: Internet Information Services, HIS: Hospital Information System, EMR: Electronic Medical Record, CPOE: Computerized Physician Order Entry, LIMS: Laboratory Information Management System, RIS: Radiology Information System, PACS: Picture Archiving Communication System, ERP: Enterprise Resource Planning.

The four selected apps have the same technical architecture (Figure 5). We implemented a broker server for mobile apps to enforce the security of clinical data in legacy system. This broker server prohibits direct access to the legacy clinical database by mobile client application. It provides Secure Socket Layer (SSL) and data encryption function, and Jolt-based secure communication channel to the legacy database [23]. Finally, the captured clinical data stored in the legacy database pass through the data control layer. The mobile client application comprises of following five layers: Data input layer, Presentation layer, Business layer, Data layer, and Network layer. Clinical data from sensors on smartphone captured and handled by above five layers. Then captured clinical data were sending to broker server.

## 3. Results and Discussion

### 3.1. My Cancer Diary

**Figure 6:**
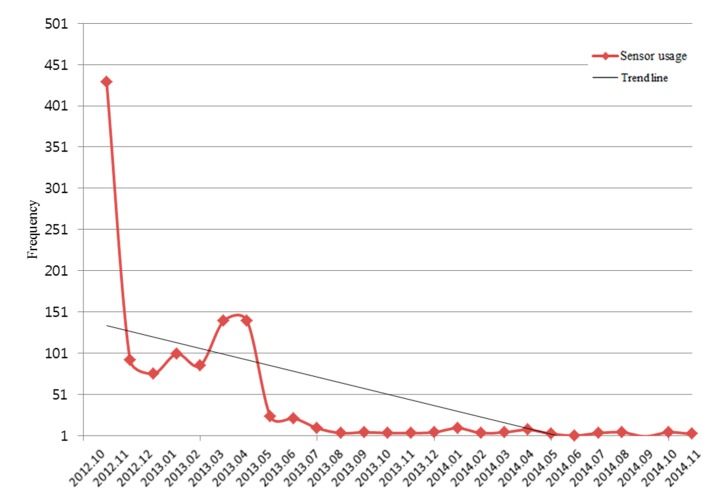
Frequency of sensor usage in “My Cancer Diary” by month.

The “My Cancer Diary” app uses the smartphone camera for patients’ log-in by recognizing the patient’s barcode or QR code. This app was downloaded 7960 times via Google Play and installed in 2483 devices. The app was downloaded 2094 times via the iTunes App Store during this period (the iTunes App Store only provides the data since June 2013). Over 26 months, the sensor for “My Cancer Diary” was used 1194 times. The usual login by ID/password (PW) was used 56,151 times during the same period. Only 2% of logins used the barcode or QR code reader. Figure 6 shows the frequency of sensor usage by month with a trend line. The sensor usage by patients dramatically declined during the first three months. After six months, most patients shifted to use the conventional ID/PW method. We expected that an easy login using a camera scan would be beneficial for users; however, the results imply that the simple login capability with the camera scan only was not preferred by users.

### 3.2. Point-of-Care HIV Check App

**Figure 7:**
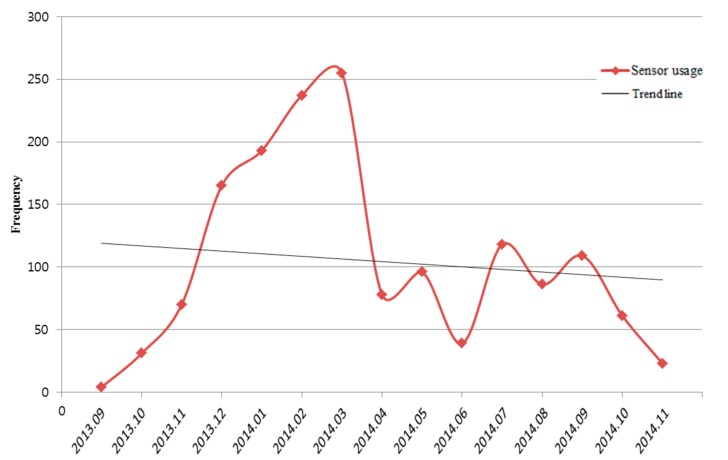
Usage pattern of Point-of-Care HIV check app by month.

The “Point-of-Care HIV Check” app identifies the patient’s ID by a barcode and takes a photo of the test results using the smartphone camera. This app was released to 10 clinical nurse specialists in the emergency room [20]. This app was used 1565 times over 14 months. During this period, six HIV-infected patients were detected. Although this app demonstrated its benefits, the overall trend of usage declined slightly (refer Figure 7). The app was actively used during first six months; however, usage rapidly declined in April, 2014 when the charger of devices in the emergency room was out-of-order. This hardware problem was immediately resolved, but the app usage did not subsequently recover. Even though this app can provide a clear benefit to users, users regard the use of this app as an additional burden to their routine jobs.

### 3.3. Blood Culture App

**Figure 8:**
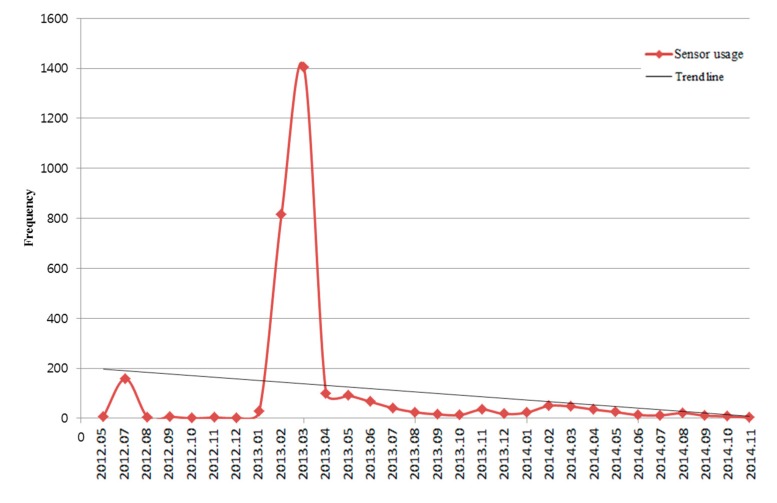
Frequency of usage of the “Blood Culture” app by month.

The “Blood Culture” app identifies the patient’s barcode using the iPhone camera and creates a timestamp for the blood culture. This app was released to four intern doctors at the pilot stages. And then it was released to all intern doctors in AMC (1568 persons). Up to now, 372 intern doctors have used this app. The app was used 3076 times over 29 months. When we looked at the log data of this app (Figure 8), there was one big peak and two minor peaks in the accumulated number of logins per month. One minor peak was in July 2012, around the pilot study of its practical usage. During the three-week pilot phase from 4 July 2012 to 26 July 2012 in the medical intensive care units, 356 sample data were collected by four intern doctors. Other peaks occurred from February 2013 to March 2013 and from February 2014 to March 2014. During February and March, most new trainees start their work rotation at the hospital. Considering the function of this application to guide the proper method for blood culture, this pattern of usage seems to be affected by the cycle of alterations of manpower and education for newcomers. However, the usage declined after one or two months.

When those interns evaluated the “Blood Culture” app, they evaluated this app as necessary for patient safety, patient and sample identification, and timeliness. Interestingly, one intern complained about the many data entry fields. However, this app was regarded as good for patient safety, identification, timeliness, and efficiency. This app could acquire important information about the sampler, sample time, sample site, and sample volume at the point of blood sampling without a delay from clinical processes.

### 3.4. mAMIS

**Figure 9:**
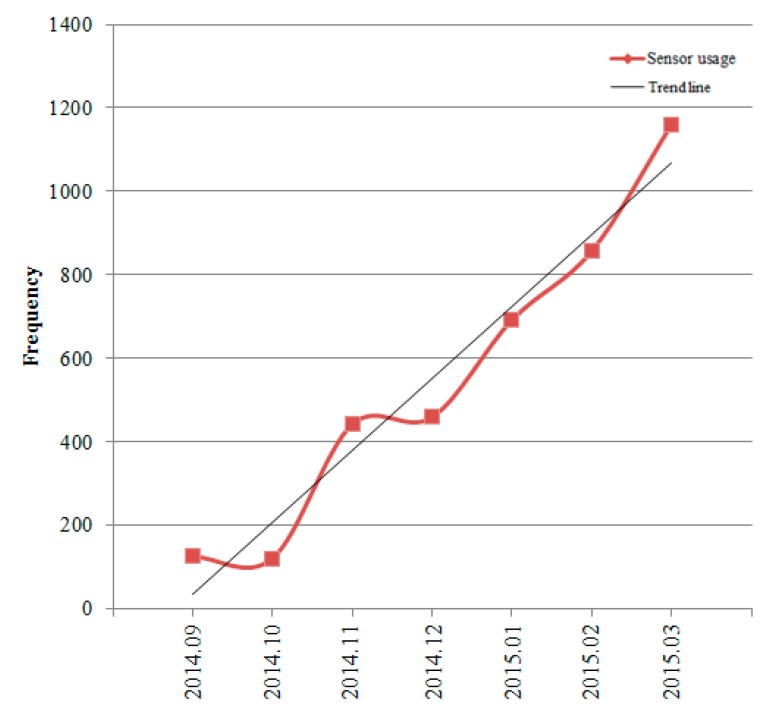
Frequency of usage in mAMIS app by month.

The mAMIS app was released to all physicians and nurses (5688 persons) in our hospital. Among them, 3004 persons used mAMIS. After the release of the upgraded mAMIS, the Touch ID login was used 3856 times during seven months. The usual ID/PW logins were used 250,129 times for the same period. Only 1.54% of logins on average used the Touch ID sensors. The rate looks still quite low compared to the ID/PW method. However, if we consider that only 38 users (1.3%) among total 3004 mAMIS users used Touch ID to login, the ratio seems to be reasonable. Interestingly, the usage pattern in Figure 9 steadily increased unlike those of the other apps analyzed. This implies that if mobile sensors can guarantee convenience or a workload reduction, users will voluntarily use mobile sensors.

### 3.5. Analysis of Usage Patterns of Healthcare Apps with Smartphone-Based Sensors

**Figure 10:**
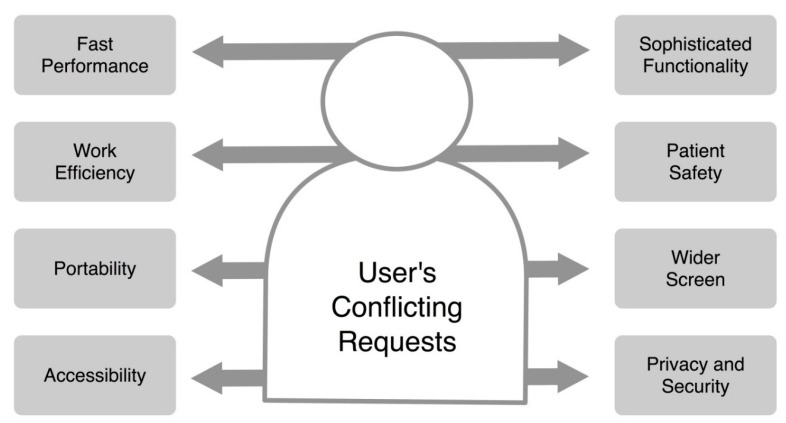
The conflicting requests of healthcare app users. Arrows indicate the trade-off relationships.

Most of the four apps analyzed here were heavily used at the early stages of implementation or for some specific periods, but subsequently were not actively used. Though there could be many reasons for these declines in usages, and we suspect the following reasons: first, when launching new apps, the hospital actively promoted their usage by introducing them to users or by starting a pilot study. However, these promotion activities were suspended after a period of a few months. If the clinical activities or processes of user were not aligned with the usage of the developed apps, users started to reduce usage due to the additional steps required or the lack of a significant benefit to users as in the case of “Point-of-Care HIV Check” and “Blood Culture”. This pattern implies that the usage of smartphone apps in a tertiary hospital is affected by the system or platform of the hospital, while other healthcare apps are chosen by personal demand. Hence, the apps with mobile sensors should be communicated or integrated into the hospital information system. Second, despite all the merits of smartphone apps with sensors, physicians may consider using them to be an additional skill or task that they have to practice in addition to an already demanding workload, not reducing the existing workload. Therefore, if this becomes inconvenient for some reason before users reach the plateau of the learning curve, the application might be abandoned [24]. The interaction with the user and updates of app functions at the AMC are not sufficient to address this inconvenience. As we can see in the case of “Point-of-Care HIV Check” app (Figure 7), a drop-off in usage was caused by a minor dysfunction of the devices or apps, and this could not be recovered. Third, users request conflicting features for healthcare apps as shown in Figure 10. Users want a device that is light-weight with a wider screen and operates quickly, but also supports sophisticated functions that usually run on a PC. Users also want to use the apps anywhere, anytime, and with a high security level. All these features exist in a trade-off relationship. That’s why the usage rate of mobile apps for data entry drops quickly. At the early stage, users tried to use the data entry feature due to curiosity or the policy of the hospital. However, they reduced to use the feature because of those trade-offs.

## 4. Conclusions and Outlook

Currently, the technology for mobile sensors in our hospital is far from mature and there are still technological issues to be addressed. These include: the inconvenience of mobile devices (small size of the window and difficulties entering characters), instability of the application, and low network speed and performance. There will also be challenges for building a data processing strategy in the hospital, because such sensor data represent a new type of information that has not been used before. The lack of a proper clinical procedure also needs to be resolved, such as requiring nurses to follow the laborious steps to use barcode system. Another major problem is the lack of a standardized format for sensor data. For instance, diverse barcode formats for patient identification are used in various departments. There also are potential security problems in the wireless network and applications that need to be tackled in relation to the deployment of mobile sensors in health.

However, despite any potential hurdles, it is likely only a matter of time before smartphone sensors are actively used in the hospital setting because healthcare apps with sensors can be used to monitor, warn, and provide health information. For example, the current mobile sensors, such as a portable pulse oximeter, have been used during the waiting time for examinations or at the time of transfer. However, the current portable pulse oximeters cannot record or send the data from patients to the EMR. Therefore, mobile devices connected to smartphones for monitoring vital signs that could be used universally by both expert and less experienced users, may reduce the blind spots in monitoring and recording [25]. In critically ill patient, those devices might provide a prompt alarm to the physician via the EMR system. Besides, if wireless devices for vital sign monitoring can be universalized, they may reduce the workload of health care providers to check and record vital signs manually. A smartphone with a camera can be a tool to record, monitor and communicate [26]. As we mentioned above, the smartphone camera can automatically send the images to the EMR system. In addition to the case in this paper, the photos of a skin lesion in the department of dermatology or the videos of neurologic examinations in the department of neurology may provide much more objective information than statements from physicians. However, when we use ordinary cameras for medical recording, it is troublesome not only because of its inconvenience, but also because of the security problems, caused by the use of memory cards to store the images. Because a QR code can be easily printed and quickly decoded, the QR code can be used for decision support system by containing the pharmacogenomic data of patients [27]. In a tertiary hospital, if we put the alert data of patients (e.g., bleeding tendency or hypersensitivity to specific drugs) into QR codes, health providers could receive critical information without needing to use a desk computer. A QR code alert system may serve to reduce the adverse events that happen during administration of drugs, examinations, or interventions, particularly, because sensors such as a barcode or QR code reader can provide fast and correct links to the EMR system. For this convenience, the main role of smartphone sensors may be as a bridge between users and the EMR system, especially during the periods of adaptation. However, QR codes have not been used actively in other industries [28]. Therefore, we need to find out the correct use cases of mobile sensors and educated the users to guarantee correct usage of sensors.

In addition, attachable sensors for smartphone can greatly expand the range of application [29,30]. As the size of medical devices has been getting smaller, the potential usage of smartphones will expand, not only as transmitters connecting to the EMR system, but also for a portable data-processing system. Traditionally, portable devices for diagnosis were regarded as preliminary methods before confirmative examination. However, palm-sized diagnostic devices are available now which have similar power as the enzyme-linked immunosorbent assay (ELISA), and can respond within fifteen minutes [29]. This allows smartphones with add-on sensors to provide medical care of higher quality to patients who cannot easily access a hospital, and even to the patients in unpredictable disasters [30]. In a tertiary hospital with more complex patient situations, these add-on sensors could increase the efficiency by increasing the discontinuation of the flow of medical care. Eventually, the cost of laboratory tests could be saved and the workload of healthcare providers might be reduced.

The potential of smartphones, amplified by various built-in and add-on sensors is limitless. Though there could be many trial-and-errors similar to our hospital’ cases, smartphones with accompanying sensors may play their own distinct role in the medical field with their unique function and flexible usage. In this study, the graphs of frequency of usage against time were given to demonstrate the usage patterns. The development of the relevant metrics should be investigated to provide more insights into why the usage patterns decreased with time.
